# Effect of Needling Parameters and Manufacturing Porosities on the Effective Thermal Conductivity of a 3D Carbon–Carbon Composite

**DOI:** 10.3390/ma12223750

**Published:** 2019-11-14

**Authors:** Abdulrahman Alghamdi, Hamzah Alharthi, Ali Alamoudi, Abdullah Alharthi, Ammar Kensara, Scott Taylor

**Affiliations:** 1 Mechanical Engineering Department, Umm Al-Qura University, Makkah 24382, Saudi Arabia; haharthi@uqu.edu.sa (H.A.); ali.alamoudi.2015@gmail.com (A.A.); ammar.kensara@gmail.com (A.K.); 2 WMG, University of Warwick, Coventry 24382, UK; Scott.Taylor.1@warwick.ac.uk

**Keywords:** needle-punching, carbon–carbon composite, thermal conductivity, finite-element modelling

## Abstract

Needle-punching is used as an alternative to expensive and sophisticated three-dimensional (3D) weaving processes to prepare a 3D composite. In this study, a 3D needled carbon–carbon (C/C) composite structure was examined using X-ray tomography and scanning electron microscopy (SEM). The effects of manufacturing porosities, needling diameter and needling density on the thermal conductivity of the composite were determined through multiscale finite-element modelling. The degradation of thermal conductivity caused by the manufacturing porosity was higher in the longitudinal direction than in the through-thickness direction. Moreover, it was found that the through-thickness thermal conductivity of the composites increased with increasing needling diameter and density.

## 1. Introduction

Carbon–carbon (C/C) composites are widely used in the aerospace and nuclear industries due to their superior properties, such as low density, high corrosion resistance, high thermal conductivity with low coefficient of thermal expansion and good frictional performance [1,2,3]. Therefore, these composites are extensively used in aircraft brake systems [4] and as nozzle throat inserts in solid rocket motors [5,6].

Two-dimensional C/C composites have low interlaminar strength and low through-thickness thermal conductivity. Enhancement of these properties requires reinforcing fibres in the direction perpendicular to the lamina (the through-thickness direction). The processes for weaving three-dimensional composites are sophisticated and expensive. However, three-dimensional needle-punching technology can overcome these limitations [7,8]. In this technique, unidirectional cloths and short-fibre felts are stacked in alternating layers. The stacks are punched with hook-fitted needles to create three-dimensional structural preforms [9]. Complex shape preforms can be produced quickly and cheaply through needle-punching. However, many parameters of the needling processes, such as needling diameter, density, distribution, and depth may vary with the high speed of the needle-punching process, which results in changes to the properties of the composite. The influence of these structural parameters on the mechanical properties of the composite was investigated in previous studies [9,10,11,12]. A considerable amount of research has also been conducted to investigate the influence of different types of manufacturing porosities on the effective thermal properties of two-dimensional weave composites [13,14,15,16,17,18].

However, although the three-dimensional (3D)-needled C/C composites are used in high-temperature applications, neither the effect of needling parameters nor manufacturing porosities on the thermal properties of a composite has been investigated. Therefore, this study aims to determine the effect of manufacturing porosity and needling parameters on the thermal transport properties of a 3D-needled C/C composite.

## 2. Materials and Methods

### 2.1. Material under Investigation

The investigated composite was fabricated by Beijing Great Wall Co., Ltd. (Beijing, China). It consists of 10 layers of 0° and 90° piles of unidirectional cloth stacked alternately, and separated by short-chopped fibre felt. The average thicknesses of the unidirectional cloth and the short-chopped fibre felt are 0.38 mm and 0.204 mm, respectively. The short-chopped fibres are distributed randomly in the felt. The preform was obtained by punching these layers with hook-fitted needles. Finally, the preform was infiltrated with liquid resin, followed by pyrolysis to convert the resin into a carbon matrix. Multiple cycles of impregnation were then used to densify the composite.

### 2.2. X-ray Computed Tomography

A Nikon Metris X-ray micro-tomography instrument at the Manchester X-ray Imaging Facility was used to acquire raw 3D tomographic data on the investigated composite. In X-ray tomography, the examined specimen is irradiated with an X-ray beam. A detector measures the X-ray intensity after transmission through the specimen. Many factors influence the transmission of X-rays through the sample, such as X-ray energy, the specimen size and material properties. The imaging parameters are listed in Table 1. 

### 2.3. Laser Flash System

Two disk specimens were cut from the composite, one in the longitudinal direction and other one in the through-thickness (transverse) direction. Thermal diffusivities of both specimens were measured by using a laser flash system (Model LFA 427, NETZSCH, Selb, Germany). It works on the principle proposed by Parker et al. [19]. A laser pulse was applied on the entire front face of a disc specimen for a few milliseconds, while temperature changes were recorded on the back face of the specimen as a function of time. From the recorded data, thermal diffusivity was calculated using the equation:

(1) $$\alpha =0.1388\times \frac{L^{2}}{t_{1/2}}$$

where *α* is thermal diffusivity, *L* is the thickness of the disc specimen and *t*_1/2_ is half-rise time, which is the time required to reach half of the maximum temperature.

Thermal diffusivity can be used to calculate thermal conductivity (*k*) from the equation:

(2) $$k=\alpha \cdot \varrho \cdot c_{p},$$

where *ρ* and $c_{p}$ are the specimen’s density and specific heat, respectively.

### 2.4. Finite-Element Modelling Details

A multiscale finite element modelling approach has been employed to predict the longitudinal and through-thickness thermal conductivity (*k*) of the composite. All the finite-element (FE) modelling was carried out using the ABAQUS^®^ software package (6.14, Dassault Systèmes Simulia Corp. Johnston, RI, USA) [20].

#### 2.4.1. Unidirectional Cloth Model

**Figure 1:**
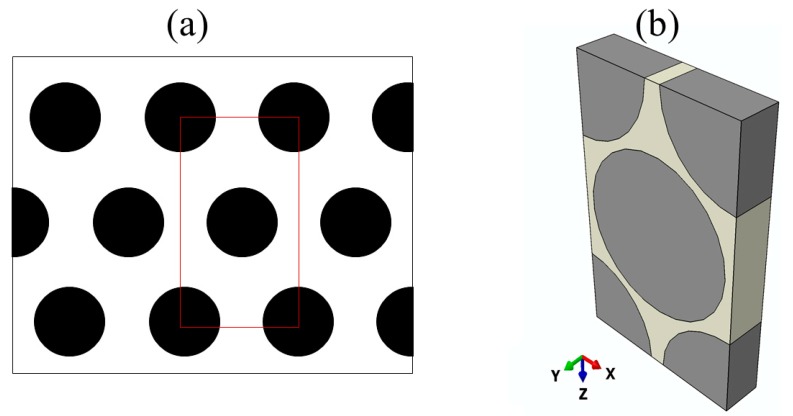
Unidirectional cloth: (**a**) side view with a hexagonal distribution of fibres, and (**b**) finite-element (FE) model of the unidirectional cloth.

The unidirectional cloth model consists of carbon fibres aligned in one direction and encapsulated by a carbon matrix. Figure 1a shows an illustration of a cross-section of unidirectional cloth with a hexagonal carbon fibre array. Because of the periodic feature of the fibres, a unit cell was constructed as the FE model of the unidirectional cloth, as shown in Figure 1b. This model contains 80% carbon fibre and 20% carbon matrix. The finite-element mesh of this model consists of 13,911 nodes and 70,892 linear tetrahedral elements of type DC3D4. The thermal conductivities of the unidirectional cloth were predicted in both the longitudinal (parallel to the fibre) and through-thickness (perpendicular to the fibre) directions. The material properties assigned to the carbon fibre and carbon matrix in this model were taken from Table 2. 

#### 2.4.2. Short-Chopped Fibre Felt Model

**Figure 2:**
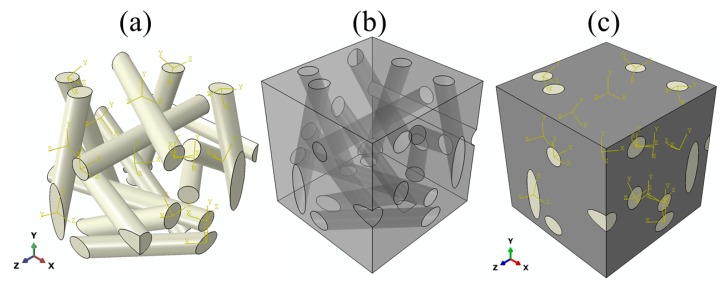
Short-chopped fibre felt model showing (**a**) carbon fibres, (**b**) carbon matrix and (**c**) the entire model.

The felt has a random distribution of short-chopped fibres, unlike the unidirectional cloth, which has periodically packed fibres. Therefore, a representative volume element (RVE) for the felt was constructed, as shown in Figure 2. The felt RVE was constructed carefully to be statistically representative of the real felt microstructure. Also, due to the anisotropic properties of the carbon fibres, each fibre in the felt RVE was given a unique local coordinate system depending on the longitudinal direction of the fibre, as shown in Figure 2a. The felt RVE consists of 16% short-chopped carbon fibres and 84% carbon matrix. The finite-element mesh of this model consists of 90,025 nodes and 453,790 linear tetrahedral elements of type DC3D4. The material properties assigned to the carbon fibre and carbon matrix in this model were taken from Table 2.

#### 2.4.3. Composite

**Figure 3:**
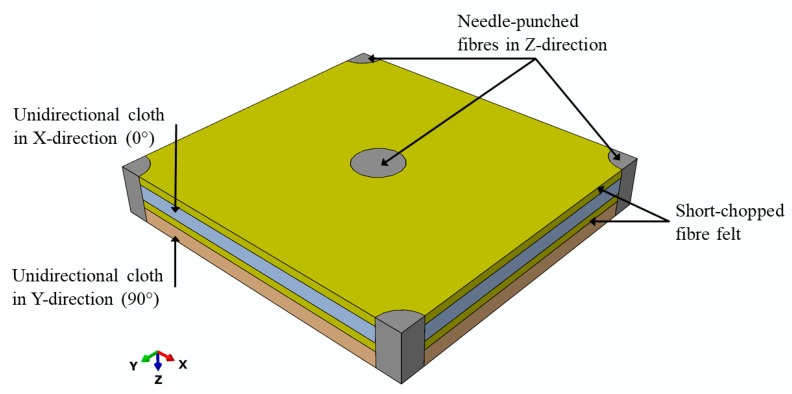
The unit cell of the composite.

The composite unit cell was constructed based on the observations of the scanning electron microscopy (SEM) and tomographic images of the composite microstructure. Figure 3 shows the unit cell of the composite. The constructed unit cell contains four layers: two layers of chopped-fibre felt separated by two layers of unidirectional cloth, one oriented in a 0° and the other one in a 90° direction. The needle-punched fibres were assumed to have cylindrical shapes going through these four layers.

The composite unit cell has one needling yarn in the centre of the unit cell, in addition to one quarter of a needling yarn on each corner of the unit cell, giving a total of two needling yarns per unit cell. Similar unit cells were used in different studies [12,21] to model needled composites. It was also assumed that the needling yarn has the same fibre–volume fraction as the unidirectional cloth, and therefore the same thermal properties, but in different directions.

#### 2.4.4. Boundary Conditions

Boundary conditions are created by applying a temperature gradient (temperature difference) on the two opposite parallel planes, while the remaining faces are insulated. These boundary conditions create a heat flow going in one direction towards the cold surface. Thermal conductivity in that direction (*k*) is calculated using Fourier’s law:

(3) $$k=-\frac{Q}{A}.\frac{L}{∆T},$$

where *Q* and *A* are the total heat flux and the area of the cold surface, respectively, *L* is the distance between the two parallel planes across which the temperature gradient is applied, and ∆*T* is the temperature difference across the model.

#### 2.4.5. Material Properties of the Constituents

The input thermal properties and densities of carbon matrix, carbon fibre, and air are given in Table 2.

## 3. Results

### 3.1. Manufacturing Porosities

**Figure 4:**
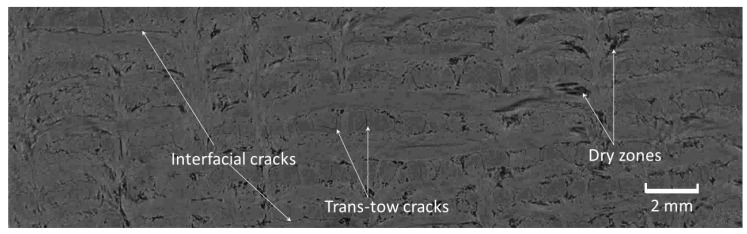
Tomographic image of the composite, showing different types of porosity.

Several studies have classified the manufacturing porosities of ceramic composites into three main groups: trans-tow cracks, interfacial cracks and dry zones [14,16,17]. The same types of porosity can be observed in the tomographic image of the examined composite, as shown in Figure 4.

**Figure 5:**
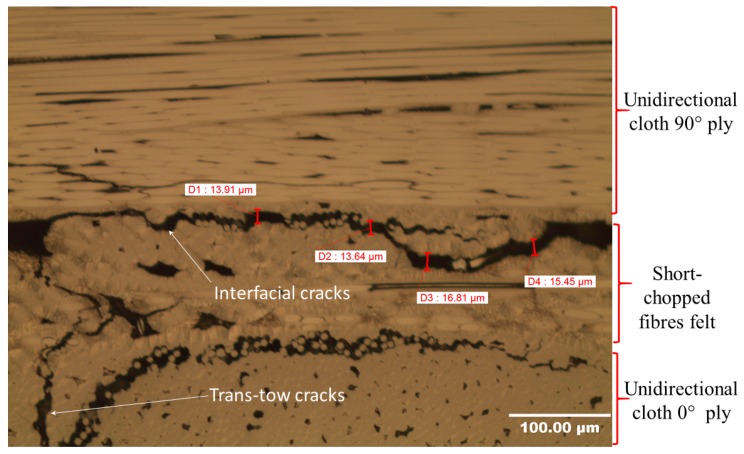
Scanning electron microscopy (SEM) image showing trans-tow and interfacial cracks.

Trans-tow cracks are periodic cracks that run through the unidirectional cloth (0° and 90° piles) parallel to the direction of fibres. Several authors [13,25,26] have found that these cracks result from the thermal stresses generated during the heat-treatment process. These cracks have semi-regular spacing between them. The average value of spacing between these cracks, as well as the crack width, were measured at higher resolution using SEM and were found to be 0.39 mm and 7.9 µm, respectively. Figure 5 shows a trans-tow crack, and an interfacial crack that partially separates between the unidirectional cloth and the short-chopped fibre felt. It was also generated from thermal stresses as a result of matrix shrinkage [25,26].

**Figure 6:**
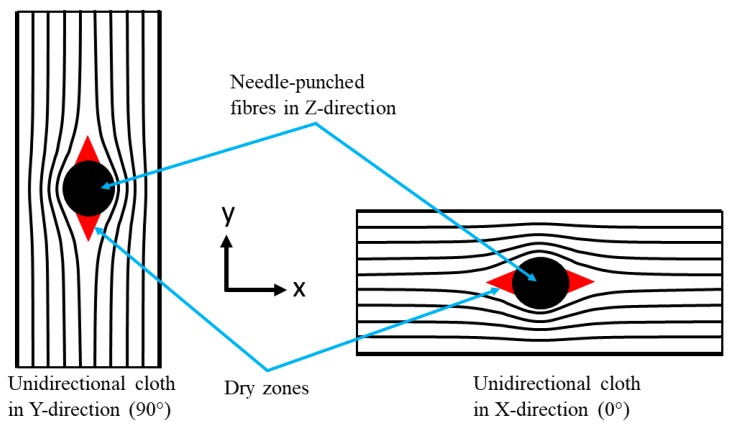
Schematic showing the locations of dry zones in 0° and 90° unidirectional cloths.

**Figure 7:**
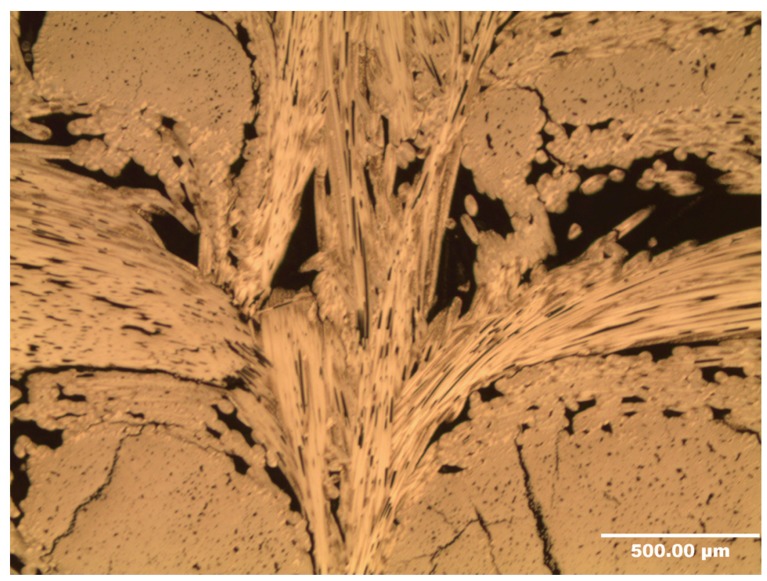
SEM image of the needle-punched fibres, surrounded by a dry zone.

Dry zones comprise large voids which occur in the unidirectional cloths around the needle-punched fibres. Figure 6 shows a schematic diagram of the dry-zone areas in the unidirectional cloth layers. The absence of a carbon matrix in these dry zones results from incomplete infiltration of the liquid resin, or inappropriate cure and carbonisation cycles [4]. Figure 7 shows an SEM image of dry-zone areas around the needle-punched fibres.

### 3.2. Thermal Properties

Table 3 gives three experimental thermal diffusivity values for each specimen, obtained using the laser flash system. The mean values of longitudinal and through-thickness thermal diffusivity were used in Equation (2) to obtain thermal conductivity values. The longitudinal and through-thickness thermal conductivities were found to be 48.62 W/(m·K) and 22.48 W/(m·K), respectively. These values were taken as reference values to validate the thermal finite-element predictions of the thermal conductivity of the composite.

### 3.3. FE Modelling

The followed strategy was used to determine the thermal conductivities of the unidirectional cloth and short-chopped fibre felt first, by constructing a finite-element model for each layer. The results from both models were then assigned to two different homogenous materials representing these layers in a composite unit cell. The thermal conductivity result of this composite unit cell was compared with the experimental data of the laser flash system for validation purposes in the following section.

The predicted thermal conductivities of the unidirectional cloth in the longitudinal and transverse direction were found to be 88 and 14.34 W/(m·K), respectively. Thermal conductivities of the felt RVE were predicted in all three directions (X, Y and Z). The results for the short-chopped fibre felt model are shown in Table 4.

#### 3.3.1. Effect of Manufacturing Porosity

**Figure 8:**
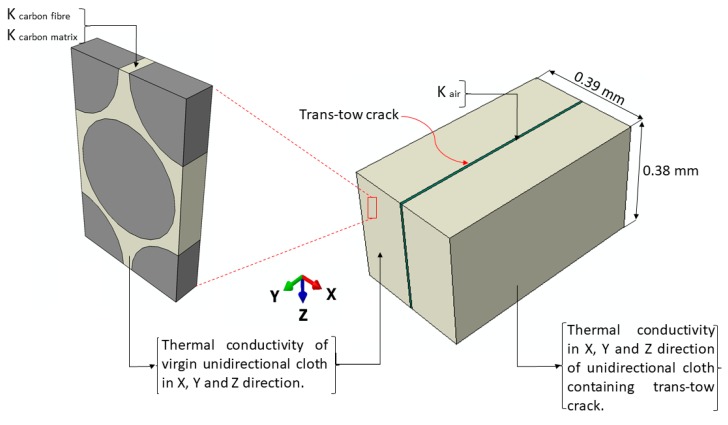
Multiscale modelling of thermal conductivity of a unidirectional cloth containing a trans-tow crack.

The degradation of the thermal transport properties of the unidirectional cloth, due to the presence of trans-tow cracks, was considered by constructing a three-dimensional model of a homogenous unidirectional cloth containing a trans-tow crack. The width of this model is equal to the average trans-tow crack spacing, which is 0.39 mm, as determined in Section 3.1. The width of the trans-tow crack in this model is 7.9 µm, which is the average value of the trans-tow crack width. The properties of air were assigned to the crack. Thermal conductivities of the virgin unidirectional cloth, found earlier in the unidirectional cloth model (Figure 1b), were assigned to the rest of the model. The results were incorporated into the unidirectional cloth layers in the composite unit cell. Figure 8 shows the adopted multiscale modelling technique for the thermal conductivity of the unidirectional cloth containing trans-tow cracks. The thermal conductivities of this system were found to be 10.8, 87.06 and 14.19 W/(m·K), in the X, Y and Z directions, respectively.

**Figure 9:**
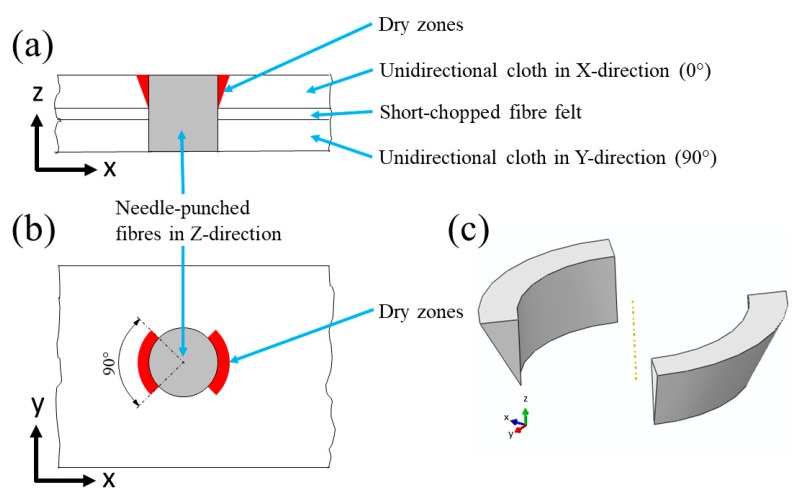
A simplification of the dry zone structure: (**a**) front cross-section and (**b**) top view, (**c**) three-dimensional (3D) structure of the dry zone.

**Figure 10:**
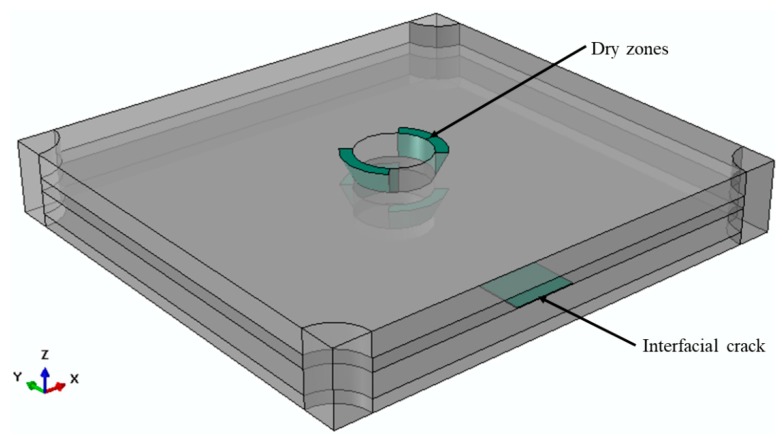
Transparent composite unit cell showing dry zones and an interfacial crack.

Dry zones were simplified and modelled as right-angled triangles rotated by 90° around the needle-punched fibres, in the same direction as the fibres in the unidirectional cloth layers. Figure 9 shows a schematic of the simplification of dry zones in the composite unit cell. Figure 9a shows a front cross-section of a needle-punched fibre with a dry zone in the unidirectional cloth in the X-direction. Figure 9b shows the top view of the needle-punched fibre. The dry zones are located in the first and the third quarters, around the needle-punched fibres. In the unidirectional cloth in the Y-direction (90°), the dry zones are located in the second and the fourth quarters, around the needle-punched fibres. Figure 9c shows a three-dimensional representation of the dry zone in one layer. Interfacial cracks were also included in the composite unit cell as thin layers partially separating the unidirectional cloth from the short-chopped fibre felt, as shown in Figure 10.

Table 5 shows the thermal conductivity values of the composite unit cell with and without porosity in both the through-thickness and longitudinal directions. The degradation in thermal conductivities was predicted to be 1.5% and 4% in the through-thickness and longitudinal directions, respectively. 

The measured and predicted thermal conductivities of the composite are shown in Table 6. The predicted values are 7–10% lower than the experimental values. This may be associated with the assumption of an idealised composite unit cell. Neglecting the matrix-rich regions in the composite in the FE modelling could have been another reason for this reduction. Nevertheless, the finite-element results obtained from the composite unit cell used in this study are in good agreement with the experimental results.

#### 3.3.2. Effect of Needling Diameter

**Figure 11:**
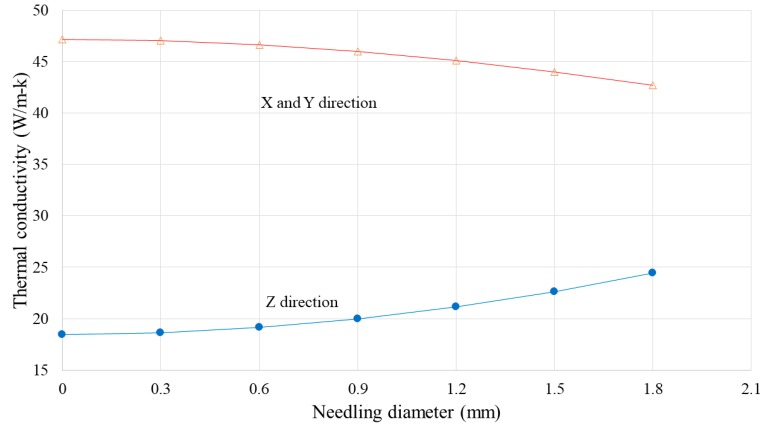
Composite thermal conductivity as a function of needling diameter.

In order to investigate the effect of the needling diameter on the effective thermal conductivity of the composite, the composite unit cell was constructed six times with different needling diameters. Steady-state boundary conditions were applied to all the models in the longitudinal (X and Y) and through-thickness (Z) directions in order to determine the thermal conductivities in each case. As evident from the results shown in Figure 11, increasing the needling diameter reduces the thermal conductivity of the composite in the X and Y directions and increases the through-thickness thermal conductivity in the Z direction. However, thermal conductivity has an exponential relationship with the needling diameter.

#### 3.3.3. Effect of Needling Density

The influence of needling density was investigated by constructing multiple composite unit cells with fixed needling diameter (1.2 mm) and different surface areas. Similar to the needling diameter, the thermal conductivity was predicted in the longitudinal (X and Y) and through-thickness (Z) directions.

**Figure 12:**
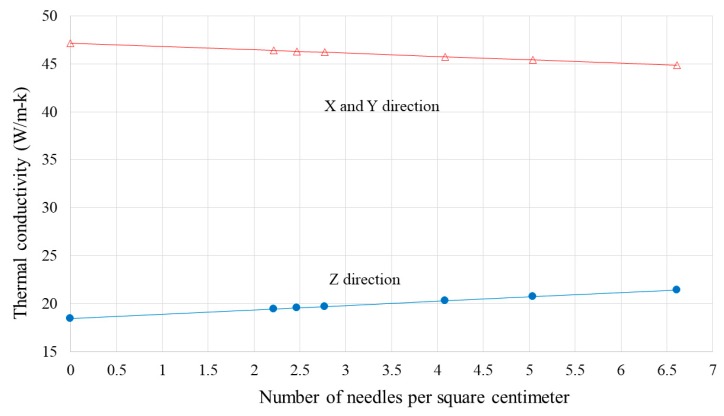
Composite thermal conductivity as a function of needling density.

As shown in Figure 12, increasing the needling density reduces the thermal conductivity of the composite in the X and Y directions and increases the through-thickness thermal conductivity in the Z direction. However, in both cases the relationship between thermal conductivity and needling density is linear.

## 4. Discussion

This is the first (to our knowledge) investigation of the thermal conductivity of needled C/C composites depending on manufacturing porosities and the characteristics of the needling process. Based on the observation of tomographic and SEM images, a multiscale modelling approach for a 3D needled C/C composite was presented. Two different models were constructed to predict thermal conductivities for the unidirectional cloth layer (with and without trans-tow crack), and short-chopped fibre felt. The predicted values of thermal conductivities from these models were utilised in a composite unit cell containing interfacial cracks and dry zones. The modelling results were in good agreement with the experimental results obtained from the laser flash test, which lends credibility to the more advanced computational results.

By modelling the heat transport in the confirmed model of a composite unit cell with and without cracks, it was found that the degradation of thermal conductivity caused by the manufacturing porosity was higher in the longitudinal direction than in the through-thickness direction. This reduction mainly caused by trans-tow cracks.

The through-thickness thermal conductivity of the composite increased with increasing needling diameter and needling density. The increase was linear for needling density and exponential for needling diameter.

The longitudinal thermal conductivity of the composite decreased with the increase in needling diameter and needling density. The reduction was linear for needling density and exponential for needling diameter.

## Figures and Tables

**Table 1:** X-ray imaging parameters.

Current (mA)	Voltage (kV)	Rotation Path (°)	Pixel Size (µm)	Exposure Time (ms)
100	50	0.3	19.4	2001

**Table 2:** Properties of the constituent materials.

Phase	Thermal Conductivity W/(m·K)	Specific Heat J/(kg·K)	Density kg/m^3^	Source
Carbon Fibre	100(//) 10(┴)	921	1800	[13,14]
Carbon Matrix	42.2	1256	1400	[22,23]
Air	0.026	1000	1.3	[24]

**Table 3:** Experimental results of laser flash tests.

Test Number	Thermal Diffusivity (mm^2^/s)	
	Longitudinal	Through Thickness
1	38.16	17.677
2	38.026	17.536
3	37.817	17.492
Mean	38.001	17.569
Standard deviation	0.173	0.097

**Table 4:** Thermal conductivity results from the felt representative volume element (RVE) model.

Direction	Thermal Conductivity (W/(m·K))
x	40.5
y	35.6
z	43.2
Average	39.76

**Table 5:** Thermal conductivities of the composite unit cell with and without porosity.

Direction	Thermal Conductivity (W/(m·K))	
	Without Porosity	With Porosity
Through-thickness	21.13	20.82
Longitudinal	45.4	43.66

**Table 6:** Comparison of the FE results with the experimental results.

	Thermal Conductivity (W/(m·K))	
Direction	Experimental	FE Modelling
Through-thickness	22.48	20.82
Longitudinal	48.62	43.66

## Nomenclature

**Symbol**	**Description**	**Units**
*α*	thermal diffusivity	mm^2^/s
*L*	thickness of the sample	m
*t_1/2_*	half rise time	s
*k*	thermal conductivity	W/(m·K)
*ρ*	density	Kg/m^3^
*c_p_*	specific heat	J/(kg·K)
*Q*	total heat flux	W
*A*	surface area	m^2^
*∆T*	temperature difference	°C
**Abbreviations**	**Meaning**	
*C/C*	carbon–carbon	
*RVE*	representative volume element	
